# A Review on Electroporation-Based Intracellular Delivery

**DOI:** 10.3390/molecules23113044

**Published:** 2018-11-21

**Authors:** Junfeng Shi, Yifan Ma, Jing Zhu, Yuanxin Chen, Yating Sun, Yicheng Yao, Zhaogang Yang, Jing Xie

**Affiliations:** 1 Department of Chemical and Biomolecular Engineering, The Ohio State University, Columbus, OH 43210, USA; shi.356@buckeyemail.osu.edu (J.S.); ma.1711@buckeyemail.osu.edu (Y.M.); 2 Department of Biomedical Engineering, The Ohio State University, Columbus, OH 43210, USA; 3 College of Pharmacy, The Ohio State University, Columbus, OH 43210, USA; syusejing@gmail.com; 4 Department of Neurosurgery, Mayo Clinic College of Medicine, Jacksonville, FL 33573, USA; chen.yuanxin@mayo.edu; 5 School of Life Sciences, Jilin University, Changchun 130012, China; sunyt16@jlu.edu.cn (Y.S.); cqupharm@163.com (Y.Y.)

**Keywords:** electroporation, microfabrication, miniatured electroporation, intracellular delivery

## Abstract

Intracellular delivery is a critical step in biological discoveries and has been widely utilized in biomedical research. A variety of molecular tools have been developed for cell-based gene therapies, including FDA approved CAR-T immunotherapy, iPSC, cell reprogramming and gene editing. Despite the inspiring results of these applications, intracellular delivery of foreign molecules including nucleic acids and proteins remains challenging. Efficient yet non-invasive delivery of biomolecules in a high-throughput manner has thus long fascinates the scientific community. As one of the most popular non-viral technologies for cell transfection, electroporation has gone through enormous development with the assist of nanotechnology and microfabrication. Emergence of miniatured electroporation system brought up many merits over the weakness of traditional electroporation system, including precise dose control and high cell viability. These new generation of electroporation systems are of considerable importance to expand the biological applications of intracellular delivery, bypassing the potential safety issue of viral vectors. In this review, we will go over the recent progresses in the electroporation-based intracellular delivery and several potential applications of cutting-edge research on the miniatured electroporation, including gene therapy, cellular reprogramming and intracellular probe.

## 1. Introduction

**Figure 1:**
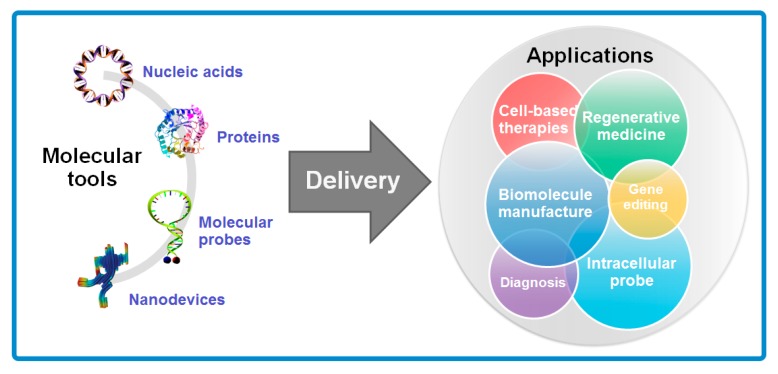
Various applications in different fields benefit from the development of molecular tools for intracellular delivery.

Cells are the basic building blocks of all living organisms, while molecules, as the currency of information in cellular exchange, are responsible for the cellular structure and orienting cells toward specific function. Many diseases such as immunodeficiencies [1], cancers [2], Parkinson’s disease [3] and Alzheimer’s disease [4] are primarily triggered by the disorder and malfunction of cells. To treat these diseases, introducing exogenous molecules and pertinent materials into cells is an important strategy in deciphering cell function, guiding cell fates, and reprogramming cell behaviors, which are expected to greatly contribute to the normal function of cells and recovery of tissues. Recently, many molecular tools have been developed to modify genes for treatment of diseases and probe intracellular microenvironment, including nucleic acids (such as DNA plasmids, mRNA, microRNA and siRNA) which can overexpress certain genes in cells for various function, inhibitor proteins as drugs for anti-tumor treatment, molecular beacon probes and DNA origami enabling investigation of intracellular environment and bio-sensors and nanodevices for manipulation at the molecular level [4,5,6]. These molecular tools render intracellular delivery broad prospect not merely in the biomedical field, in which cell-based therapies, regenerative medicines and early diagnosis are introduced, but also in the bio-manufacturing and biological fundamental research where biomolecule manufacture, gene editing and intracellular investigation are focused respectively (Figure 1).

**Figure 2:**
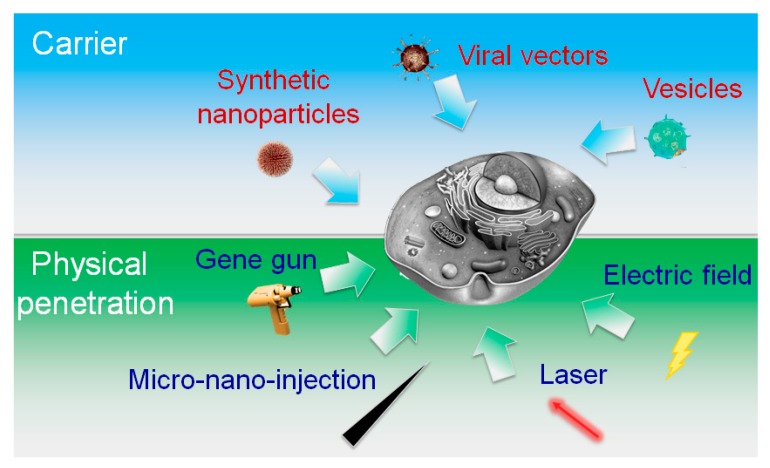
General classification of popular intracellular delivery platforms: carrier-mediated delivery strategies (blue background) and popular physical membrane-penetrating delivery methods (green background).

In the process of intracellular delivery, cargos and approaches for transportation are two key elements. Cargos for delivery are highly variable in size, sources, organization and physicochemical properties. Such diversity of cargo could make great impact on the delivery strategies and intracellular applications. Based on the diverse properties and applications of cargos, current techniques for intracellular delivery can be mainly categorized into two types: carrier-mediated delivery and membrane-penetrating delivery. As illustrated in Figure 2, carrier-mediated delivery comprises various biochemical assemblies, mostly of molecular to nanoscale dimensions, while membrane-disruption are closely related to physical effect, involving the incorporation of transient discontinuities in the plasma membrane via mechanical, electrical, thermal, optical or acoustics means.

When comes to the carrier-mediated delivery, biological and chemical strategies are principally covered, which include extracellular vehicles (EVs), viral vectors and organized nanoparticles. Viral vectors (e.g., retrovirus and lentivirus) containing packaged gene materials of interest inside can effectively target host cells and release the genetic molecules consequently. While viral transduction can achieve a high efficiency, it can inherently disrupt the immune system and thus consistently raise safety issues in vivo. For the extracellular vesicles, especially exosomes, enthusiastic attention have been concentrated on this biological field because of their important roles in cell-to-cell communication [7]. These vesicles not only provide new biomarkers for disease diagnosis [6,8,9,10,11], but also emerge as a potent delivery agent [12,13,14,15]. Despite enormous potential, the underlying mechanism of EVs delivery remains to be unveiled before widely used and extended to clinical application. Furthermore, chemical methods were developed for the delivery of sensitive genetic cargoes and drugs toward specific target, which are designed to be enclosed in liposome- or polymer-based nanocarriers that can be uptaken by cells via endocytosis [16,17,18,19,20,21]. Tremendous efforts have been made to develop novel nanoparticle formations and their therapeutic applications particularly in drug delivery [22,23,24,25,26,27,28,29,30,31]. Yet limitations to their successful intracellular delivery, a poor understanding of their interaction with biological environments, highly stochastic dosage in delivery vesicles and the toxicity issues related to these novel materials have retarded their deployment inside the cell [32].

Membrane-disruption modalities are primarily physical methods, which can be classified as direct penetration modalities and permeabilization. Direct penetration employs a solid conduit or vehicle to concurrently penetrate the membrane and introduce cargo. Prevalent samples here are mechanical cell membrane penetration (micro- and nano-injection) [33] and gene gun with ballistic particles [34] as shown in Figure 2. Unlike carrier-based delivery, active external force is utilized for puncturing the cell membrane to obtain access in direct penetration, where the target cell must respond to repair damage sustained to the plasma membrane or other cellular structures. Alternatively, permeabilization strategies aim to make a cell become permeable to a substance when disruptions in the membrane are of sufficient size to allow “package” through the membrane. Almost all the permeabilization strategies need apply specific conditions, such as temperature, electric field and buffer composition, to initially motive permeabilization and delivery and subsequently facilitate cell recovery. Common commercialized tools for permeabilization are laser-based delivery and bulk electroporation. Comparing to the direct penetration strategies, permeabilization strategies enable better control of membrane disruption effect with the regulation of parameters in different conditions. Generally, a balance of the membrane disruption effect need to be found in effective permeabilization strategies, optimizing both the membrane damage and cell treatment conditions.

Among several choices in permeabilization-based disruption delivery, electroporation has already been an established technique in medical field, but many of its biotechnological applications, especially intracellular delivery did not start to emerge until 1980s [35]. Due to its feasible control and efficiency in cell transfection and desirable delivery for a huge variety of cargos from small molecules to larger proteins/antibodies, electroporation has been deemed as one of the most promising methods and widely utilized for intracellular delivery. Although widespread applications, some challenges, such as precise dose control and cell viability remains to be demonstrated. In recent years, with the development of nanotechnology and microfabrication, lab-on-chip, microfluidic, and nanotechnological systems have emerged for the revolution of miniatured electroporation, which to great extent addressed the challenges in permeabilizing cells initiated by electric field. 

This review will mostly focus on the electroporation-based intracellular delivery, especially the combination with novel technologies, extended miniatured electroporation and its potential applications in clinic. We will first cover the brief history and mechanisms of electroporation. These include an overview of development of electroporation and principles of traditional electroporation system, as these characteristics are inextricably linked to the deficiencies and challenges involved in intracellular delivery. We then survey some of the most promising technical improvement in electroporation, especially in miniaturized electroporation. Technical advances will be compared with traditional electroporation system and potential clinical applications will be introduced. 

## 2. Overview of Electroporation

### 2.1. Brief History of Electroporation

**Figure 3:**
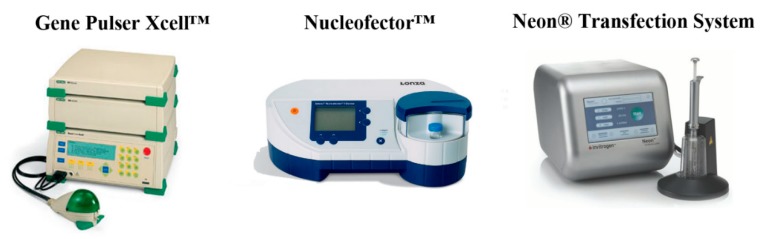
Commonly-used commercial bulk electroporation (BEP) system. From left to right: Gene Pulser Xcell™ from Bio-Rad, Nucleofector™ from Lonza, and Neon^®^ Transfection System from Thermo Fisher Scientific. Figures from Internet. copyright by the companies.

The interest of people in applying electricity in human and animal bodies could date back to 1700s, and it was found that electric field caused damage to the tissues presumably due to the irreversible electroporation [36]. Systematic study of electroporation at the cellular level, however, did not begin until 1980s. In nucleated mammalian cells, Neumann and his colleagues published a groundbreaking report in 1982, which demonstrated that electroporation could give rise to the efficient transfection of plasmid DNA in mouse lyoma cells [35]. This study also pioneered electroporation theory by introducing a novel model to simulate the extent of permeabilization in an electroporated cell with a generalized van’t Hoff relationship, whereby poration phenomena could be viewed as structural rearrangements of lipids and water. In 1990s, Tsong published a series of research papers which were considered milestones in understanding the fundamental biophysics of electroporation technology and its applications. The definition of electroporation given by Tsong which is still widely accepted now is that electroporation is the transient loss of semi-permeability of cell membranes subject to the electric pulses, thus leading to “ion leakage, escape of metabolites, and increased uptake by cells of drugs, molecular probes, and DNA” [37,38]. After entering the 21st centuries, electroporation has been one of the most popular non-viral cell transfection methods both in vitro and in vivo, which could be attributed to the maneuverability of the system and its versatility in terms of transfection cell types. Due to the prominent advantages of electroporation in cell transfection, such technology has been applied in biomanufacture, and common commercial electroporation systems are listed for instance in Figure 3.

### 2.2. Physical Principles of Electroporation

**Figure 4:**
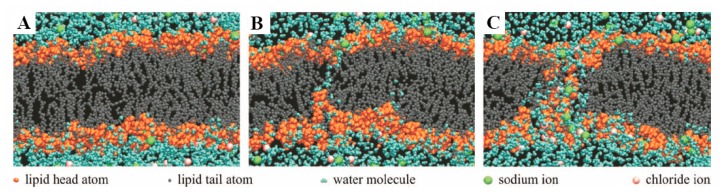
Molecular dynamics showing the progress of an aqueous pore forming within the lipid bilayer during electroporation. From left to right (**A**) the intact bilayer, (**B**) a few water molecules enter the lipid regime, starting to form a “water path”, and (**C**) the neighbouring lipids reorient, stabilizing the “water pore” and allowing the ions to enter. Reprinted with permission from ref. [39] Copyright © 2012, IEEE.

Cell membrane is known to be basically composed of a lipid bilayer with thickness about 5 nm, which functions as a barrier to the cellular components from extracellular environment. As an electrical insulator exhibiting excellent dielectric property in normal physiological conditions, it maintains the electric potential (~0.07 V) across the membrane due to significant difference of ion concentration between cytosol and the fluid in extracellular microenvironment. Theoretically, electroporation is an effective strategy to form pores in cell membrane by the application of a potential difference across that membrane. When the potential difference reaches a specific magnitude of voltage, the probability of electroporation taking place on cell membrane drastically increases. In actual, the electric breakdown of cell membrane happens when the transmembrane potential across the lipid bilayer $\Delta_{m}$ reaches a threshold, reportedly a critical value about 1 V [37], because the lipid molecules within the membrane re-orient to form small hydrophilic openings (“aqueous pathways”) on the cell membrane, which is otherwise hydrophobic in the undisturbed state (as shown in Figure 4). This breakdown can be either reversible or irreversible, depending on the electric pulse intensity and duration as well as the cell types.

A variety of factors have been studied to model the transmembrane potential $\Delta_{m}$ Schwan equation is one of the most widely-used models to calculate $\Delta_{m}$, as shown below:

(1) $$\;\Delta_{m}=-f\cdot E\left(t\right)\cdot R\cdot cos\theta \cdot \left(1-e^{-\frac{t}{\tau }}\right)\;$$

where f is the cell-shape factor (1.5 for spherical cells), E is the applied external electric field, R is the radius of the cell, θ is the polar angle between the direction of E and the specific location on the cell membrane, *t* is the time, and *τ* is the time constant of the cell membrane “capacitor” (characteristic charging time ~1 μs). Therefore, in the steady-state condition, τ≪t, and the equation above can be simplified into:

(2) $$\;\Delta_{m}=1.5\cdot E\cdot R\cdot cos\theta \;$$

This equation is feasible for the estimation of $\Delta_{m}$ for the electroporation in different cell types. Recently, with the rapid development of numerical simulation, sophisticated models with a much larger set of parameters were developed to more accurately predict the electric field distribution and $\Delta_{m}$ on single cells [40,41,42]. In addition to the transmembrane potential associated with electrical conductance, physicochemical, thermal, and electromechanical membrane deformation effects may also contribute electroporation. The application of mechanical tension has been demonstrated to significantly abate the electric voltage threshold required for membrane disruption. This could be ascribed to the bias of energy landscape when defect forms. Similar with the effect of mechanical tension, lower temperatures are reported to increase the electric field strength required for electroporation and further slow the kinetics of resealing of cell membrane. Although many of mathematical descriptions and simulated models have been developed to assess the effect of external parameters mentioned above on the deformation of cell membrane, challenges are remained to verify in actual application. 

### 2.3. Bulk Electroporation in Cell Suspensions 

**Figure 5:**
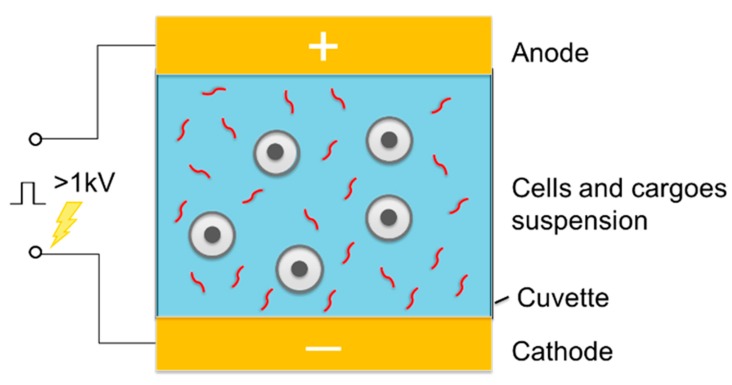
Schematic of electroporation system for cell suspensions.

Electroporation has been widely applied to deliver a diverse range of cargo molecules and materials of interest into the intracellular space. Conventional electroporation technique for the intracellular is done in cuvette-style parallel plate setups, where the cell suspension and molecules to-be-delivered are mixed together in the conducting buffer solution between two electrodes connected to a generator of high electric voltage, and thus it is called bulk electroporation (BEP). In such a BEP setup (Figure 5), an approximately homogeneous electric field could be obtained across the cell suspension. From the aspect of suspended cells in the cuvette, upon application of voltage, different region of the plasma membrane of cells could reach the trans-membrane threshold potentials with different time, which results in growth of a heterogeneous distribution of pores over the cell surface. Due to the inherent negative potential of cells, permeabilization tends to occur first at the hyperpolarized side of the cell facing the positive electrode with more numerous pores over membrane of the cells, while the pores of cells formed on the depolarized side may carry larger pores in diameter but with less amount [43,44]. Generally, the coverage area of the permeabilization in bulk electroporation is primarily controlled by pulse strength, while the overall pore size is more associated with the pulse duration [43]. Based on such heterogeneous permeabilization, the concrete response within different cell populations various with properties of cells such as cell size, membrane composition and physiological condition, as well as variances of applied electric filed as mentioned. These factors have been investigated in detail by artificial model and theoretical calculation because of the significance of cell viability after electroporation-based intracellular delivery. However, lack of experimental methods to measure the realistic effect has made that still challenging to validate [45].

On the strength of permeabilization triggered by electric filed, bulk electroporation has been used to deliver a diverse range of cargo molecules and materials of interest to the intracellular space. These cargos range from small molecules to proteins and macromolecules, including dyes, impermeable drugs, molecular beacons, proteins, siRNA, DNA plasmid and nanoparticle. As expected, intracellular delivery mediated by bulk electroporation is influenced by not merely the pore diameter, but also the cargo dimensions as well as the charge of the cargo molecule. For small molecules, diffusion across concentration gradient throughout the duration of a pore’s lifetime is the main route [46]. If the molecules are charged, such as propidium iodide, which carries two positive charges, there is an added electrophoretic component that can augment delivery during the pulse [47,48]. As for the proteins and macromolecules, these larger molecules exhibit a narrow window of opportunity to enter cells when comparing to the small molecules [43]. In this process, large pores may shrink almost instantly after turning off the electric filed while the small pores may linger in the plasma membrane for minutes [48]. Thus, pulse timing, especially longer pulse duration is more efficient strategy for the intracellular delivery of larger molecule. In addition to the cargo size, charge of the cargo molecule also plays a key role in influx mechanisms of electroporation. Unlike the diffusion of small molecules, the mechanisms of large molecules in the electroporation-based delivery, particularly charged macromolecules such as nucleic acid, are regarded to be almost entirely dependent upon electrophoretic forces provided during the pulse [43,46,49]. In general, longer pulse durations, more prominent aggregation of charged molecules around cell membrane, and higher delivery and cell transfection efficiency could be eventually achieved.

Taken together, BEP in cell suspension is mainly determined by the pore diameter and the cargo properties. During BEP, transient electrical pulses are applied to create intense electric field across cell membrane and induce the permeabilization of cell membranes. Meanwhile, the biomolecules are simultaneously delivered inside (or extracted from) the cells. For a portion of cells, because they are inevitably exposed to an extremely high electric field, the membrane disruption become irreversible which gives rise to the failure of membrane repair or prolonged apoptotic responses. Apart from damage to the cell, delivery mechanisms of BEP are diffusion dominated, and for large transfection agents such as nucleic acids, entry into the cytosol is affected through an electrophoretic aggregation and further attachment onto the outside of the cell membrane followed by an endocytosis-like process, which to some extent renders BEP low efficiency in intracellular delivery. Nevertheless, it is worth noting that precise dose control has not been demonstrated using BEP.

## 3. Miniaturized Electroporation 

The early generation of electroporation system, especially BEP, has been widely applied in intracellular delivery because of its technical simplicity, fast delivery and almost no limitation on cell type and size. However, the cuvette bulk style with cargo suspension has its problems. As mentioned above, sharp reduction in cell mortality caused by large uncontrollable membrane disruption greatly impairs the efficiency of intracellular delivery. Moreover, this cuvette style electroporation is difficult to gain command of the precise dose of cargo delivered into target cells, which to some extent hampers the future clinical application of intracellular delivery. With the development of nanotechnology and microfluidic fabrication, electroporation is moving toward the concept of miniaturization, which largely solves the challenges in conventional electroporation. Below we will discuss the innovations that have been developed in the field of miniaturized electroporation, including micro-/nanoelectroporation and microfluidic-based electroporation.

### 3.1. Microeletroporation (MEP) and Nanoelectroporation (NEP)

**Figure 6:**
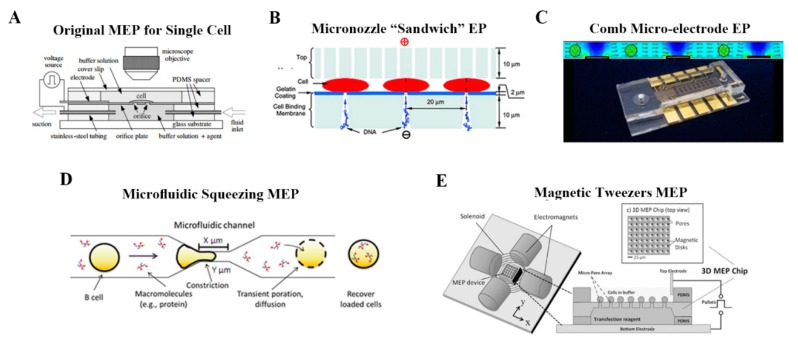
Representative schematics of micro-scale cargo delivery systems. (**A**) Electroporation through a micro-fabricated orifice to the measurement of single cell response to external stimuli. Reprinted with permission from ref. [50]. (**B**) High-throughput micronozzle-based sandwich EP system. Reprinted with permission from ref. [51]. (**C**) Flow-through comb electroporation device for delivery of macromolecules. Reprinted with permission from ref. [52]. (**D**) Microfluidic cell squeezing method. Reprinted with permission from ref. [54]. (**E**) Magnetic tweezers-based 3D MEP for high-throughput gene transfection in living cells. Reprinted with permission from ref. [55].

Recent years have witnessed a rapid progress on the development of microscale platforms for achieving electroporation at single cell level. The original MEP raised by Kurosawa’s group [50] aimed to investigate single cell response to external stimuli. It was reported that MEP showed significant advantage over BEP in terms of controllable feeding of foreign substance into a cell at a controlled timing to virtually any cells (Figure 6A). Out of the more controllable delivery, researches began to concentrate on the gene transfection as well as drug delivery in a large batch. Lee’s group [51] utilized the concept of MEP to design a membrane sandwich electroporation system through a well-defined micronozzle array, and they found higher efficiency of delivery, more uniform gene transfection and better cell viability than conventional BEP. In such MEP systems (Figure 6B), since the electric field is precisely controlled and focused in the microscale channels, individual cells that are trapped within the micro-aperture or that are flowing through the microchannel can be electroporated using a much lower voltage. Taking advantages of microchannel of MEP systems, a series of MEP-based systems of intracellular delivery have been extended. With the aid of microfluidic, Jensen’s group [51,52] created devices of MEP for improving the delivery of siRNA and proteins into HeLa cells and B cells. A novel high-throughput magnetic tweezers-based three-dimensional MEP system was also designed by Lee’s group [53]. Combination with the weak external magnetic fields, such MEP system was supposed to realize high throughput transfection and retention of cell viability. Despite the several benefits of MEP systems mentioned above, precision dosage control has not been achieved yet as it shares the similar delivery mechanism with BEP which is based on diffusion and endocytosis-like uptake.

**Figure 7:**
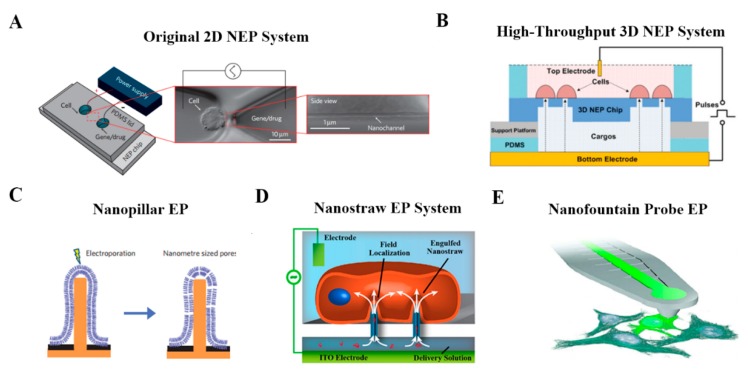
Representative schematics of nano-scale cargo delivery systems. (**A**) First-generation Nano-electroporation (NEP) device platform comprised of nanochannel array and optical tweezer for cell loading. Reprinted with permission from ref. [56]. (**B**) Motivated by limited throughput (i.e., <200 cells) of the proof-of-concecpt NEP system, high-throughput NEP platform, which features a massive nanochannel array in the z-direction handle up to 1 million cells, were developed. Reprinted with permission from ref. [60]. (**C**) Nanopillar EP. Reprinted with permission from ref. [57]. (**D**) Nanostraw EP. Reprinted with permission from ref. [58]. (**E**) Nanofountain probe (NFP) technology based on electroporation and AFM nanotip injection. Reprinted with permission from ref. [59].

Inspired by the deficiencies in early works on micro-scale electroporation, Lee’s group further introduced the concept of nanochannel electroporation [56]. As shown in Figure 7A, by scaling the aperture size down to about 90 nm, the membrane disruption effect of electroporation could be concentrated onto a very small region on the cell surface. A significant claim of this strategy is precise dose control and high cell viability, which have not been achieved by any other methods. Such nanochannel-based electroporation also appears to introduce agents faster and deeper into the cytoplasm. Compared to conventional electroporation (BEP and MEP) and other branch forms of electroporation, it is proposed that the nanochannel delivery mechanism was based on electrophoretic forces rather than diffusion and/or endocytosis. One weakness of the method, however, is the low throughput nature of the technique, in which cell to be transfected need to be positioned in one microchannel using optical tweezers. For this shortcoming, a silicon-based high-throughput NEP device was developed for high-throughput precise and benign NEP cell transfection (Figure 7B). This NEP-based biochip is capable of simultaneous transfection of ~1 million cells per ${\mathrm{cm}}^{2}$ for a single batch. Semiconductor cleanroom micro-/nano-fabrication techniques are used for engineering the nanochannel array in the z-direction of the silicon wafer. The potential biomedical applications of high-throughput NEP platform also have been demonstrated in following practical use, such as cancer cell intracellular biomarker measurement, anti-tumor drug screening, cell reprogramming, etc. In addition to the NEP platform system, there are also other forms of nanoscale electroporation such as nanopillar [57], nanostraws [58] and nanofountain probe (NFP) [59]. As for the nanopillar (Figure 7C), Cui’s group introduced a novel nanoscale system based on vertical nanopillar electrodes, which can not only record both the extracellular and intracellular action potentials of cultured cell with excellent signal strength and quality, but also repeatedly switch between extracellular and intracellular recording by nanoscale electroporation and resealing processes. Notably, subtle changes in action potentials induced by drugs that target ion channels also can be detect by these vertical nanopillar electrodes, which is very promising in the application of cell screening when targeted cells are under external stimuli. Another form of nanoscale electroporation takes the form of so-called nanostraws (Figure 7D). Unlike the NEP system mentioned before, nanoscale aperture of nanostraws protrudes into the target cell as a hollow nanoneedle, which reveals that active forces, such as optical tweezers or dielectrophoresis, are probably not required to establish optimal contact between cells and the nanostraw. Moreover, Espinosa’s group introduced a scanning probe-based approach for localized electroporation, termed nanofountain probe electroporation (Figure 7E). An atomic force microscope cantilever engineering with a hollow channel for fluid flow was designed. By coordinating the movement of the tip and the flow of fluid, molecules of interest could be delivered into cells with high transfection efficiency (over 95%), qualitative dosage control, and very high viability (over 90%) of transfected cells. 

### 3.2. Microfabrication of Miniatured Electroporation Device

**Figure 8:**
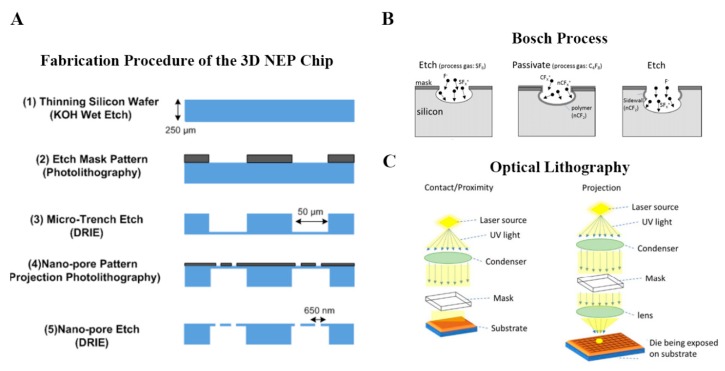
Microfabrication of miniaturized electroporation. (**A**) The fabrication procedure of the silicon 3D NEP chip. (**B**) Schematic of Bosch Process, deep reactive-ion etching (DRIE) progression. (**C**) Schematic of different types of optical lithography. Reprinted with permission from ref. [60].

As discussed in previous section, novel miniatured electroporation devices are basically micro/nanoscale lab-on-a-chip platforms, which largely relies on nanotechnology, microfluidic and microfabrication techniques. The fabrication of these micro/nanoscale electroporation devices often involves the standard semiconductor cleanroom-based wafer process steps, i.e., lithography, etching, deposition, etc. In many cases, the small features created in silicon master were replicated or transferred to polymer-based elastomeric materials, most notably polydimethylsiloxane (PDMS). Taking the fabrication of high-throughput 3D NEP chip for instance [60], the nanochannel array on the silicon substrate of the NEP platform was fabricated in a class 100 cleanroom following the protocol in Figure 8A. After a pre-preparation of wafer, optical lithography was used to print the nanopore array from the pre-designed micropore pattern on a photomask. Basically, as illustrated in Figure 8C, contact/proximity lithography imprinted equal proportion of the pattern size on the photomask into the sample, while projection lithography reduced the pattern on the photomask (or reticle) by adding an objective lens below and projecting the pattern on a small portion of the sample known as “die”. Multiple exposure is usually needed in projection lithography to cover the entire wafer with an array of dies. For the structured nanochannel in 3D NEP system, a deep silicon structure etching method, deep RIE (DRIE) “Bosch Process”, was utilized to etch a high-aspect ratio (>20:1) nanochannel array (10 µm nanochannel in depth). An alternating sequence of the etching processes (SF6 gas) and the sidewall passivation steps (C4F8) enables fast etch rate, a nearly 90° sidewall profile, and high-aspect ratio features (Figure 8B). After the fabrication of nanochannel array, a backside microchannel array was then made for the connection of nanochannel, which allowed for the function of 3D NEP. In such microfabrication of miniaturized electroporation chip, pertinent parameters, including exposure time for optical lithography and cycle of etching, are critical to achieve a well functional miniaturized electroporation system. By regulating these parameters and sequence of microfabrication procedure, various styles of lab-on-chip could be designed for the development of miniaturized electroporation in disparate applications. 

### 3.3. Cell Manipulation Techniques for Miniaturized Electroporation

Since the electric field in electroporation, which accelerates the charged transfection agents and prorates the cell membrane, could diminish exponentially outside nanochannels, it is critical to achieve close contact between the to-be-transfected individual cells and the corresponding nanochannels. A variety of cell manipulation techniques were integrated with miniaturized electroporation system to achieve accurate cell trapping against nanochannel. Optical tweezer (OT) is a precision instrument in which a focused laser beam can manipulate the dielectric microscopic particle by a piconewton-level force. Owing to the conservation of momentum, the refraction of light path across the particle which essentially is equivalent to the change of the momentum of photons, will in return provide a force on the particle. OT has been broadly used in biophysics research for force-extension measurement of biomolecules. As a particle manipulation tool, OT has been proven to be successfully implemented with the first-generation NEP device (Figure 7A) and it can achieve position of the cell against the nanochannel under the microscope for NEP transfection in a precise manner [56]. Even though OT can achieve uncoupled nanoscale displacement and positioning accuracy in all three x-y-z-directions, most OT systems provide only one laser beam and thus it can only manipulate one cell at a time. The cell loading time in the OT platform is proportional to the cell number, considering the operation of each cell is fixed. In addition, there is a upper limit of the cell number, because the previous loaded cells will gradually drift away from the nanochannel after a certain amount of time without exerted pushing force. Therefore, OT is only appropriate for low-throughput platform in which <20 cells can be carried in one batch due to the throughput limit.

To meet the cell number requirement for clinical applications, the high-throughput electroporation system requires parallel cell manipulation technique. Dielectrophoresis (DEP)-assisted cell loading method was utilized to integrate with miniaturized electroporation platform. Lee’s group [53] has demonstrated that positive DEP can parallelly translocate single cells to the nanochannel outlets in a high-throughput manner (>60,000 cells/cm^2^). In such DEP-NEP platform system, an alternating current (AC) field was used for DEP force generation which was uncoupled with AC-induced electrophoresis in NEP process. In another work of Lee and his colleagues [55], magnetic tweezer-based cell manipulation was reported to efficiently position the single cells for MEP system as shown in Figure 6E. As mentioned above, the cells labelled with magnetic micro-beads via antibody-antigen bonding can be moved to the microchannel outlet by controlled the magnetic field ready for electroporation. In addition to electromagnetic forces, cell trapping can also be achieved by the utilization of microstructure on the chip and hydrodynamic force. Recently, a simple “dipping-trap” massive cell trapping approach has been developed for high-efficiency NEP transfection, in which individual cells were mechanically trapped within the micro-trap unit. Comparing to the OT used for single-cell manipulation, techniques for high-throughput electroporation system successfully achieve mass parallelism for the intracellular delivery. In light of undesirable cell viability due to the additional requirements, such as buffer solution for dielectrophoresis [61], however, the choice of auxiliary for cell manipulation in miniaturized electroporation may need to be revised in future versions of theses combination techniques.

## 4. Potential Applications of Miniaturized Electroporation

### 4.1. Gene Therapy

Gene therapy is a simple yet revolutionary concept that treating a disease by modifying the problematic gene. Despite its potential of radically curing otherwise highly lethal disease at gene level, intracellular gene delivery strategy is one of the biggest hurdles. Miniaturized electroporation is a competitive physical gene delivery candidate, with the benefits of instantaneous delivery process, well-defined delivery dosage, high transfection efficiency, minimal cell damage and being viral-free, circumventing the inherited safety concerns posted by viral vectors [62]. Below is the discussion of electroporation-based intracellular delivery in gene therapy applications.

#### 4.1.1. Ex Vivo Adoptive Immunotherapy

**Figure 9:**
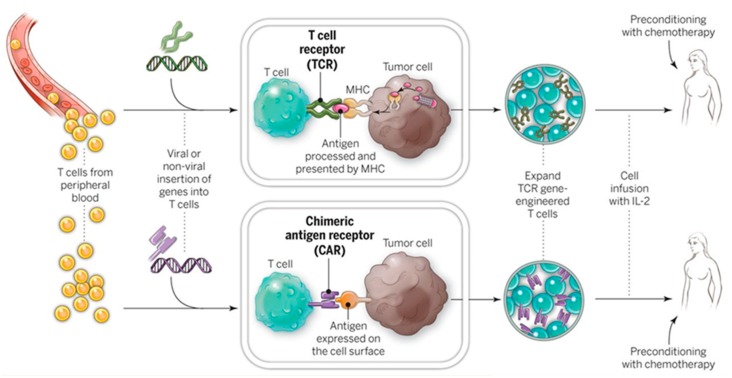
Diagram of gene therapies by personalized immunotherapy. Second row: non-viral transfection of genes into T cells is prerequisite to CAR-T immunotherapy. Reprinted with permission from ref. [65].

Genetic engineering of immune cells (e.g., T cells, NK cells) is prerequisite for adoptive immunotherapy which harnesses the patient’s own immune system to combat diseases [63,64]. As shown in Figure 9, in CAR-T therapy, patient’s T cells are transgenically engineered ex vivo, and thus express tumor-associated antigen receptors which enable the targeting of specific cancer cells in vivo [65], as illustrated in Figure 8. 

However, current technical challenge of such applications lies in the limited number of “trained” immune cells due to low yielding of plasmids transfection using conventional non-viral methods such as traditional BEP and nanocarriers [66,67,68,69]. In a recent Dielectrophoresis-assisted 3D NEP (pDEP-NEP) [61] platform was implemented for non-viral immune cell (NK-92 cell) engineering by intracellular delivery of chimeric antigen receptor (CAR) encoding plasmids and transfection efficiencies of >70% was achieve, compared with BEP with only <30%. In addition, more uniformly engineered cells (minimum cell-to-cell variability) could be provided by NEP-based transfection for potential clinical trials. Since adoptive immunotherapy requires permanent transfection of immune cells, the applicability of non-viral transient transfection methods remains a challenge. Use of less toxic yet more stably-expressed genome editing vectors [70] instead of plasmids delivered by the nanotechnology-enabled non-viral electroporation platform could solve this problem but it requires further studies. More about genome editing technology will be discussed in Section 4.1.3.

#### 4.1.2. RNA Interference (RNAi)-Based Therapy

Specific knockdown of oncogene or proto-oncogene messenger RNA (mRNA) by small non-coding RNA, e.g., small interfering RNA (siRNA) and microRNA (miRNA) has been proven an effective and secure RNAi therapeutics by a set of clinical trials including the first treatment of an RNAi therapeutic targeting VEGF and KSP in liver cancer patient [71]. Delivery of siRNA or miRNA is key to successful RNAi [71]. In previous research, lipid nanoparticles-based delivery system has been widely adopted for its good transfection efficiency, biocompatibility and ease of production [72,73]. However, it inevitably suffers from non-uniform delivery due to its stochastic process in nature. Comparing to the nanoparticles-based delivery, nanochannel electroporation (NEP)-based oligonucleotide delivery has been proven an unprecedented deterministic cell transfection method and thus a perfect technique for RNAi-based therapy which requires precision dosage control [74]. The ability to deliver siRNAs in a dose- and time-controlled manner at the single cell level allowed for the determination of optimum pro-apoptotic strategies for the potential treatment of various tumors and targeting of therapy resistant cancer stem cells (CSCs).

#### 4.1.3. Genome Editing

Manipulating the genome of the living cells by efficiently adding, deleting, or changing the DNA sequence has long fascinated biology community. In recent decades, the concept of “gene editing” has become a research “hot spot”, fueled by the breakthrough in the development of novel editing tools such as engineered nucleases. The CRISPR (Clustered Regularly Interspaced Short Palindromic Repeats)—Cas9 (CRISPR-associate protein) technology is the most widely-used platform available for gene editing because it has enormous potential to boost the development of molecular therapeutics against a variety of diseases [75]. 

Electroporation, as a common membrane-disrupted intracellular cargo molecule delivery method, has been reported to be utilized for delivering the CRISPR/Cas9 gene editing materials and thus play a profound role in gene editing [70,76,77,78]. Miniaturized electroporation (e.g., nanoelectroporation), as next-generation electroporation with the advantages over conventional bulk electroporation, is believed to potentially further spur the development of genome editing, by improving the delivery efficiency of novel gene editing molecular agents into difficult-to-transfect cell types such as immune cells and stem cells. It is recently reported that a CRISPR-Cas9 genome-targeting system by simple bulk electroporation (BEP) method has successfully reprogrammed human T cells and can rapidly and efficiently insert >1 kbp DNA sequences at specific sites in the genomes of primary human T cells while maintaining good cell viability and functionality [70]. Together with more powerful miniaturized electroporation intracellular delivery system, genome editing could open a new door for the next generation of cell-based therapies.

### 4.2. Regenerative Medicine and Cell Reprogramming

**Figure 10:**
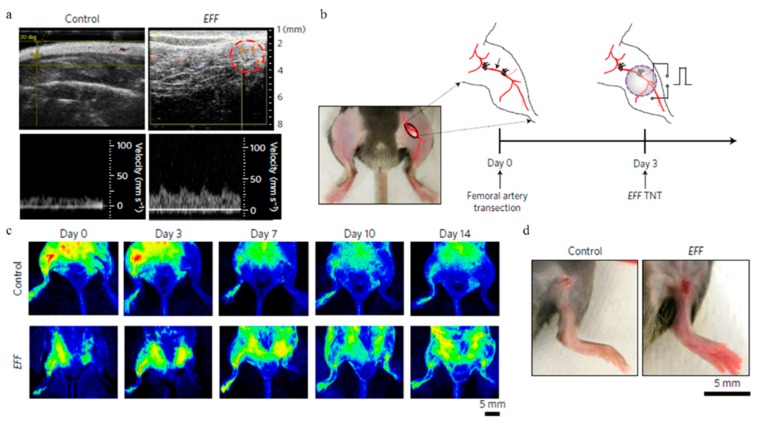
TNT-based enhanced reprogramming factor delivery and propagation can rescue whole limbs from necrotizing ischemia. Reprinted with permission from ref. [81].

Cell reprogramming can be performed on either readily available somatic cells (such as skin cells) or induced Pluripotent Stem Cells (iPSCs) [79]. Since the dosage of reprogramming factors delivered in individual cell is critical, development of the relevant tool capable of gene delivery in a deterministic manner is crucial. Lee and his group reported that an efficient reprogramming of mouse embryonic fibroblasts (MEFs) to functional neuron by a combination of three transcription factors: Brn2, Ascl1 and Myt1l (BAM), can be successfully achieved via a novel nanochannel electroporation (NEP)-based platform [80]. The induced neuronal (iN) cells generated by NEP-delivered factors could have profound effect on the future of regenerative medicine, because NEP-based iN cells could be provided fast and efficiently in a namely unlimited source of patient-specific cells of interest without the introduction of tumorigenic viral vectors. Most recently, this non-viral NEP approach (Figure 10) was implemented in vivo to topically and controllably deliver well-established and newly developed reprogramming vectors of induced neurons and endothelium (Etv2, Foxc2 and Fli1 (EFF)), respectively to tissues. The utility of this NEP based tissue nano-transfection (TNT) technology was successfully validated by rescuing necrotizing tissues and whole limbs using two murine models of injury-induced ischemia [81].

### 4.3. In Situ Intracellular Investigation

**Figure 11:**
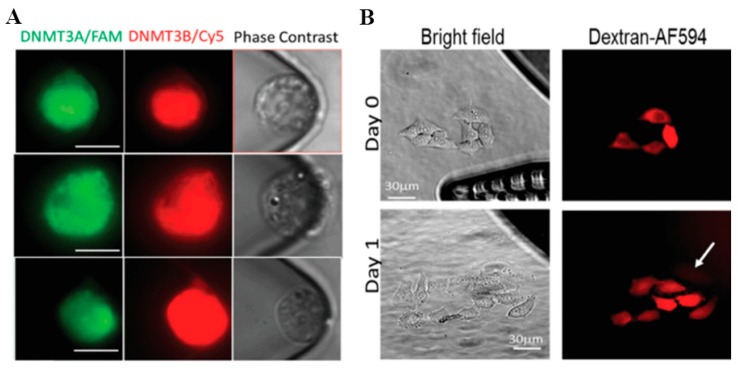
Monitor intracellular biomarker within individual living cells by molecular beacon probes. (**A**) micrographs of wild-type Kasumi-1 AML cells transfected with DNMT3A/B MBs. Reprinted with permission from ref. [85]. (**B**) HeLa cells transfected with MBs and imaged after 24 h of incubation showing that the electroporated cells divided. Reprinted with permission from ref. [86].

Living cells are complex dynamic systems that constantly interact with both intracellular and extracellular environment. Development and progression of diseases such as cancer always involve disseminating cells undergoing a variety of physically, physiologically, or pharmacologically induced events [82,83] that remains elusive using conventional cell analysis methods that are based on bulk assays and cell lysis [84]. Novel micro-/nanoelectroporation technologies enable real-time living cell measurement at single-cell level. For example, molecular beacons (MB), pre-designed oligonucleotide hybridization probes, are frequently used in intracellular cell marker detection. A nanochannel electroporation-based MB delivery platform was introduced by Zhao et at. (Figure 11A), which was used to analyze the DNMT3A/B mRNA levels of acute myeloid leukemia (AML) cells following miR-29 upregulation [85]. Giraldo-Vela et al. also used MBs, transfected into living cells by nanofountain-based electroporation, for mRNA detection at the single-cell level (Figure 11B) [86]. In vitro AXL and PDGFRα mRNA Detection by Nanochannel Electroporation (NEP)-delivered Molecular Beacon (MB) Probes was also implemented to investigate the plasticity of glioblastoma stem cells [87].

## 5. Concluding Remarks and Future Perspectives

Electroporation can deliver various range of molecular cargos into a wide variety of cell types with the precise control of pulse parameters. In this review, recent advance in electroporation-based intracellular drug delivery technology is presented with potential applications in clinic. Miniaturized electroporation, as a promising non-viral transfection method, starts to play an ever-increasingly important role in a variety of fundamental scientific research and translational clinical trials. Spatial confinement in micro-/nanoscale electroporation provides numerous benefits over conventional bulk electroporation, including rapid cargo uptake, precision dosage control and minimum cell disturbance.

Although the new generation of electroporation platforms exhibited unprecedented transfection capabilities, a large number of challenges remains to be solved. Current miniaturized electroporation still cannot transfect large enough cell population comparable to the conventional delivery methods. In other side, the transfected cells require accurate targeting to reach the disease sites in vivo for nucleic acid therapy with minimal side effects. Special expertise in micro-fabrication and instrumental operation is indispensable for the following applications. It also cannot be ignored that micro/nanoscale of the miniaturized electroporation system leads to high demand of robust fabrication protocols, which is a great challenge to biomanufacture. In view of these problems, such novel strategies have not yet been demonstrated to supersede the basic cuvette-style electroporation in clinical use.

The challenges of current electroporation systems notwithstanding, for many applications the merits outweigh the weaknesses. Instrumental progress in electroporation, together with newly-developed molecular tools, will certainly boost biomedical research from biomanufacture and clinical trials of cancer immunotherapy to ex vivo cell-based gene therapy and regenerative medicine.
